# The Century Old Taxonomic Confusion Surrounding *Wiedemannia zetterstedti* Fallén and Related Species Is Resolved (Diptera: Empididae): Revision of the *W. zetterstedti* Group †

**DOI:** 10.3390/insects13050460

**Published:** 2022-05-13

**Authors:** Marija Ivković, Bradley J. Sinclair, Emma Wahlberg

**Affiliations:** 1 Division of Zoology, Department of Biology, Faculty of Science, University of Zagreb, Rooseveltov trg 6, 10000 Zagreb, Croatia; 2 Canadian National Collection of Insects, Ottawa Plant Laboratory—Entomology, CFIA, Ottawa, ON K1A OC6, Canada; bradley.sinclair@inspection.gc.ca; 3 Department of Zoology, Swedish Museum of Natural History, 10026 Stockholm, Sweden; emma.wahlberg@nrm.se

**Keywords:** *Clinocerinae*, aquatic dance flies, new species, distribution, Europe

## Abstract

**Simple Summary:**

The aquatic dance fly species of the *Wiedemannia zetterstedti* group are studied and species definitions are re-evaluated. A new species is described from Corsica (France) and two species synonyms are proposed. The species concepts are also investigated using COI barcodes and a key to species is provided.

**Abstract:**

The *Wiedemannia zetterstedti* species group is revised after examination of all available type specimens and includes one new species (*W. ulrichi* Ivković & Sinclair sp. nov.) and four redescribed species (*W. czernyi* (Bezzi), *W. longipennis* (Mik) stat. rev., *W. rufipes* (Oldenberg) stat. rev. and *W. zetterstedti* (Fallén)). The following new synonyms are proposed: *W.* (*Roederella*) *ouedorum* Vaillant, 1952 = *W. czernyi* (Bezzi, 1905); *Paramesia riparia* Robert, 1836 = *W. zetterstedti* (Fallén, 1826). Lectotypes are designated for the following species/subspecies: *Atalanta hirtiloba* Speiser, *Brachystoma escheri* Zetterstedt, *Clinocera czernyi* Bezzi, *Clinocera longipennis* Mik, *Paramesia riparia* Robert, and *Roederia czernyi rufipes* Oldenberg. In addition to morphological evidence, molecular species concepts were investigated using a molecular phylogenetic divergence-based species delimitation (bPTP) and results confirmed the morphological conclusions. A key to species is presented and geographic distributions are mapped.

## 1. Introduction

The aquatic dance fly genus *Wiedemannia* Zetterstedt is a common empidid in streams and small rivers of Europe [1,2,3,4]. Adult flies gather on emergent rocks, where mating and feeding occurs [5]. In Europe, some 78 species are recorded, which until very recently were assigned to a number of subgenera [6]. Species of *Wiedemannia* are readily identified on the basis of facial colouration, genal width, pterostigma shape, scutal chaetotaxy and male terminalia, with their uniquely shaped clasping cercus [7,8,9]. Although the species concepts are mostly well established, species related to *W. zetterstedti* (Fallén) have remained difficult for more than a century. There are two reasons for unstable taxonomy in this group: (1) variability in the shape of the clasping cercus; (2) reluctance of workers to carefully examine the type specimens of the species/subspecies involved.

Prior to this study, there were four accepted species/subspecies in the herein named *Wiedemannia zetterstedti* group: *W. czernyi* (Bezzi), *W. czernyi rufipes* (Oldenberg), *W. ouedorum* Vaillant, and *W. zetterstedti* (Fallén). Despite the close similarity in the shape of the male terminalia, the former three species were traditionally assigned to the subgenus *Roederella* Engel and the latter to *Eucelidia* Mik on the basis of the rather weak character of absence or presence of preapical setae on the femora. The validity of the subgenera beyond Europe was questioned as early as Melander [10] and a detailed study of the subgeneric concepts was required [11]. In a molecular phylogenetic study using multiple genes, Ivković et al. [6] found most subgenera not monophyletic, and the elimination of the subgeneric classification of *Wiedemannia* was recommended. Despite this conclusion, in the Bayesian analysis [6] a group including *W. zetterstedti* and *W. ouedorum* was strongly supported.

In this study, we use both morphological and molecular evidence to test and resolve the species concepts for all members of the *Wiedemannia zetterstedti* group. This study results in the re-evaluation of past species concepts and the description of a new species. With these new species definitions, the geographic distributions are mapped.

## 2. Materials and Methods

This study is based on material borrowed from or studied in the following institutions: Canadian National Collection of Insects, Ottawa, Canada (CNC); Muséum national d’Histoire naturelle, Paris France (MNHN); Museo Civico di Storia Naturale, Milano, Italy (MSNM); Musée cantonal de zoologie, Lausanne, Switzerland (MZLS); Lund Museum of Zoology, Lund University, Lund, Sweden (MZLU); Swedish Museum of Natural History, Stockholm, Sweden (NHRS); Naturhistorisches Museum, Wien, Austria (NMW); Oxford University Museum of Natural History, Oxford, UK (OUMNH); Senckenberg Deutsches Entomologisches Institut, Müncheberg, Germany (SDEI); United States National Museum of Natural History, Washington D.C., USA (USNM); col. M. Ivković, University of Zagreb, Croatia (Ivković coll. UZC); Zoologisches Forschungsmuseum Alexander Koenig, Bonn, Germany (ZFMK); Zoological Institute, Russian Academy of Sciences, St. Petersburg, Russia (ZISP). Label data for primary types are cited from the top downward, with the data from each label in quotation marks. Labels are cited in full, with original spelling, punctuation, and date, and label lines are delimited by a slash (/). The repository of each type is given in parentheses. Secondary type data are abridged and listed alphabetically.

Photographs of pinned specimens were taken with a Leica camera model DFC5400 using Leica Application Suite X. Terms used for adult structures primarily follow those of Cumming & Wood [12]. The distribution map was made using ArcGIS Pro software (version 2.6, ESRI, Redlands, CA, USA).

To investigate the molecular species concepts in addition to the morphological studies, we used a molecular phylogenetic divergence-based species delimitation (bPTP) [13]. DNA extraction, alignment, and curation of the 658 bp barcoding region [14] was carried out according to the protocols in Wahlberg and Johanson [15]. A specimen of *Clinocera fontinalis* (Haliday, 1833) (Empididae: *Clinocerinae*) was used as the outgroup to root the phylogenetic tree during analysis. The most appropriate partition model was selected using PartitionFinder 2.1.1 [16]. Each codon position was treated separately, and possible models were limited to those available in MrBayes [17,18]. A phylogenetic tree was summarized using MrBayes 3.2.7, using the partition model scheme provided by PartitionFinder, from 50 million generations with a sampling frequency of 5000 over two runs and with a burn in of 25%. ESS and traces were checked for convergence using Tracer 1.7.1 [19]. The resulting tree was used as input tree in bPTP 0.51, with 10 million iterations, sampling frequency of 10,000, a burn in of 25%, and with the outgroup hidden from the final tree. The trace files from bPTP were examined visually for convergence. The final tree was finalized in Adobe Illustrator (version 26.0.3., Adobe Inc., San Jose, CA, USA).

## 3. Results

### 3.1. Phylogeny

**Figure 1:**
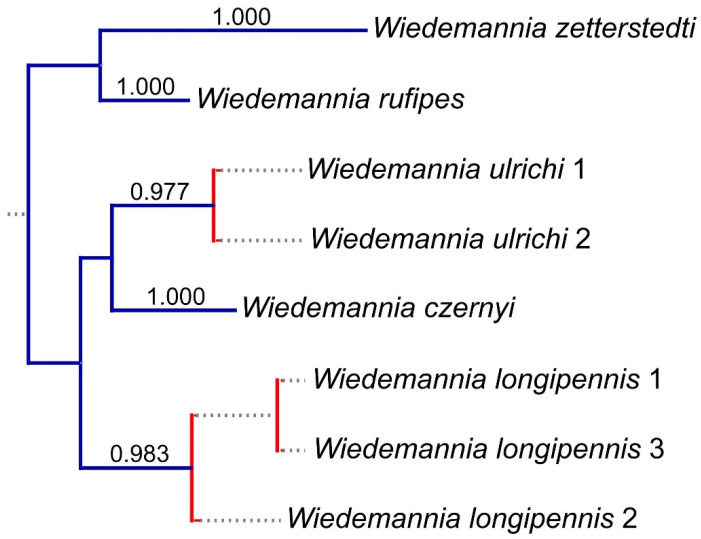
Phylogenetic tree inferred using MrBayes and the COI gene barcoding fragment, after species delimitation analysis in bPTP. Numbers above blue branches represent posterior support for each supported species, red branches are unsupported delimitations.

Based on the morphological study of the type material and additional material examined, we have concluded that the *Wiedemannia zetterstedti* group consists of five species: *Wiedemannia czernyi*, *W. longipennis*, *W. rufipes*, *W. ulrichi* sp. nov. and *W. zetterstedti*. This conclusion is also supported by the partition model scheme provided by PartionFinder dividing the codon positions individually, applying the models HKY+G (codon 1), GTR+I (codon 2), and F81 (codon 3). The resulting phylogenetic tree with suggested species is illustrated in Figure 1. The suggested species from the bPTP analysis are *Wiedemannia zetterstedti* (support = 1.000), *W. rufipes* (support = 1.000), *W. ulrichi* (support = 0.977), *W. czernyi* (support = 1.000), and *W. longipennis* (support = 0.983).

### 3.2. Taxonomy

#### 3.2.1. *Wiedemannia zetterstedti* Group

**Figure 2:**
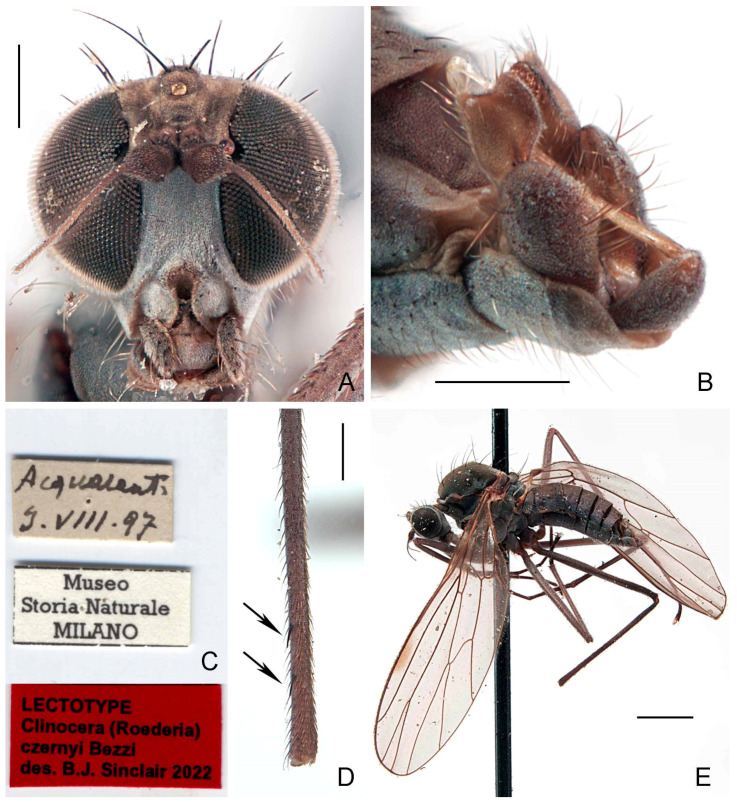
*Wiedemannia czernyi* (Bezzi). (**A**) Head, frontal view, scale bar = 0.25 mm; (**B**) male terminalia, oblique lateral view, scale bar = 0.25 mm; (**C**) lectotype labels; (**D**) hind tibia, posterior view, arrows showing erect spine-like posterodorsal setae apically, scale bar = 0.25 mm; (**E**) lectotype habitus, lateral view, scale bar = 1.0 mm.

**Figure 3:**
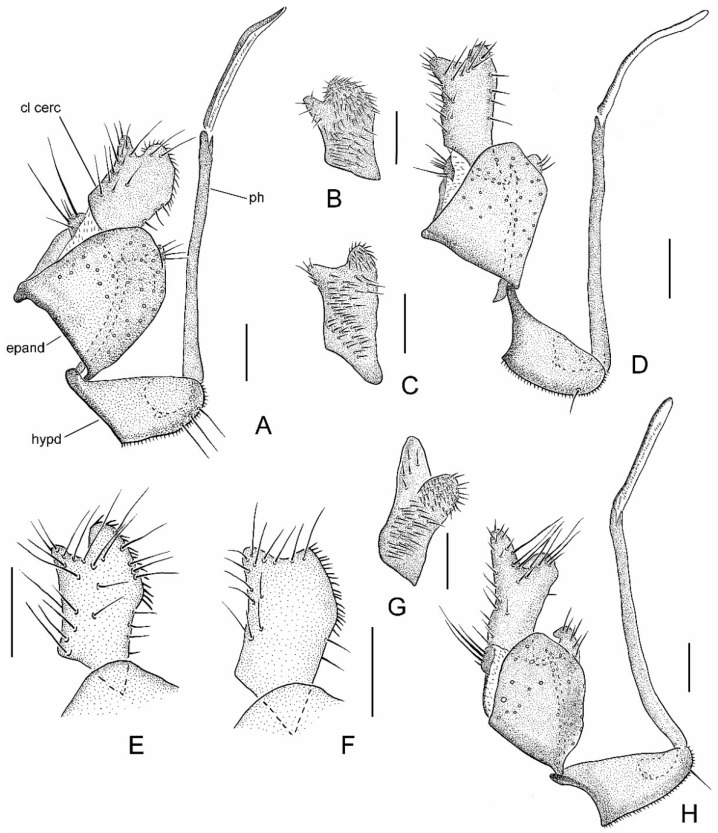
*Wiedemannia zetterstedti* group, male terminalia [setae on epandrium omitted]. (**A**) *W. czernyi*, lateral view; (**B**) *W. czernyi*, clasping cercus, inner lateral view; (**C**) *W. longipennis*, clasping cercus, inner lateral view; (**D**) *W. longipennis*, lateral view; (**E**) *W. rufipes*, clasping cercus, outer lateral view; (**F**) *W. ulrichi*, clasping cercus, outer lateral view; (**G**) *W. zetterstedti*, clasping cercus, inner lateral view; (**H**) *W. zetterstedti*, lateral view. Scale bars = 0.1 mm. Abbreviations: cl cerc—clasping cercus; epand—epandrium; hypd—hypandrium; ph—phallus.

**Diagnosis.** Gena broad, less than half as deep as height of eye; only a few small and fine, irregularly biserial presutural acrostichal setulae; fore coxae with 2–3 long, erect setae projecting beyond shorter proclinate setae (Figure 6A); pterostigma narrow, elongate and pale to brownish (Figure 2E); clasping cercus smaller than epandrium, apex bifurcated (Figure 3A–H); phallus without swelling on distiphallus.

**Remarks.**  *This group of species was previously assigned to two different subgenera, Eucelidia Mik and Roederella Engel, and included five species/subspecies: W. czernyi (Bezzi), W. czernyi rufipes (Oldenberg), W. ouedorum Vaillant, W. pirata Mik, and W. zetterstedti (Fallén) [20]. Currently, the former subgenera of Wiedemannia are no longer recognized [6] and this group of species is now referred to as the W. zetterstedti group. Wiedemannia pirata was considered related to the W. zetterstedti group on the basis of erect preapical setae on the fore femur. The former species has been removed from this species group on the basis of five pairs of dorsocentral setae, with interspersed setae and distinct biserial acrostichal setae extending around the prescutellar depression. Additionally, the clasping cercus is much larger, twice the size of the epandrium, differently shaped than in all other species of this group, and the distiphallus bears a mid-length swelling*.

#### 3.2.2. Key to Species of the *Wiedemannia zetterstedti* Group in Europe (based on dried, pinned specimens)

**Figure 4:**
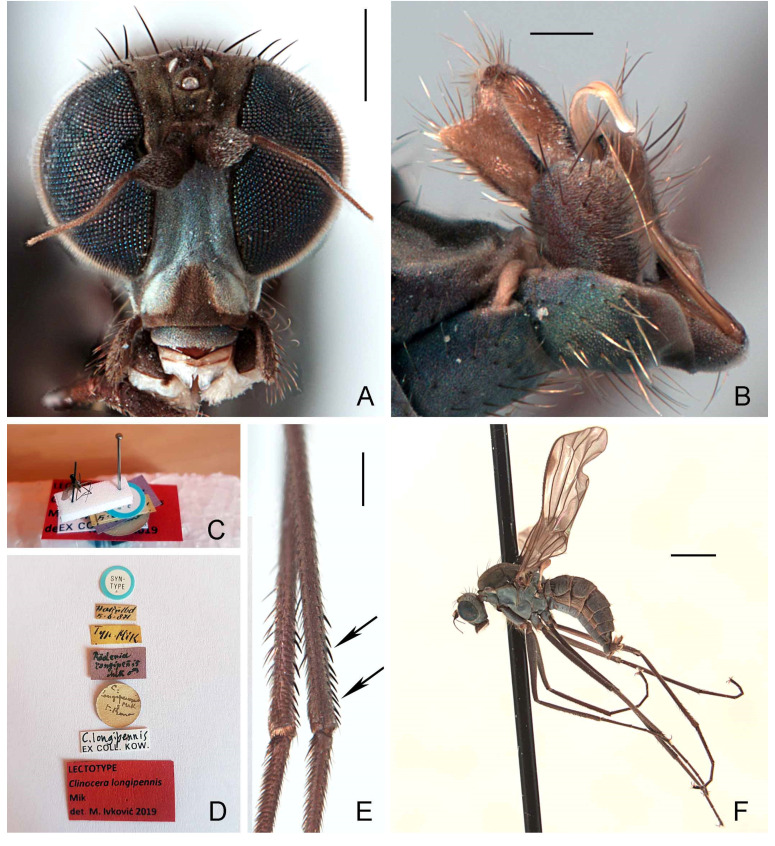
*Wiedemannia longipennis* (Mik). (**A**) head, frontal view, scale bar = 0.25 mm; (**B**) male terminalia, oblique lateral view, scale bar = 0.10 mm; (**C**) lectotype; (**D**) lectotype labels; (**E**) hind tibiae, posterior view, arrows showing spine-like posterodorsal setae, scale bar = 0.25 mm; (**F**) habitus, lateral view, scale bar = 1.0 mm.

**Figure 5:**
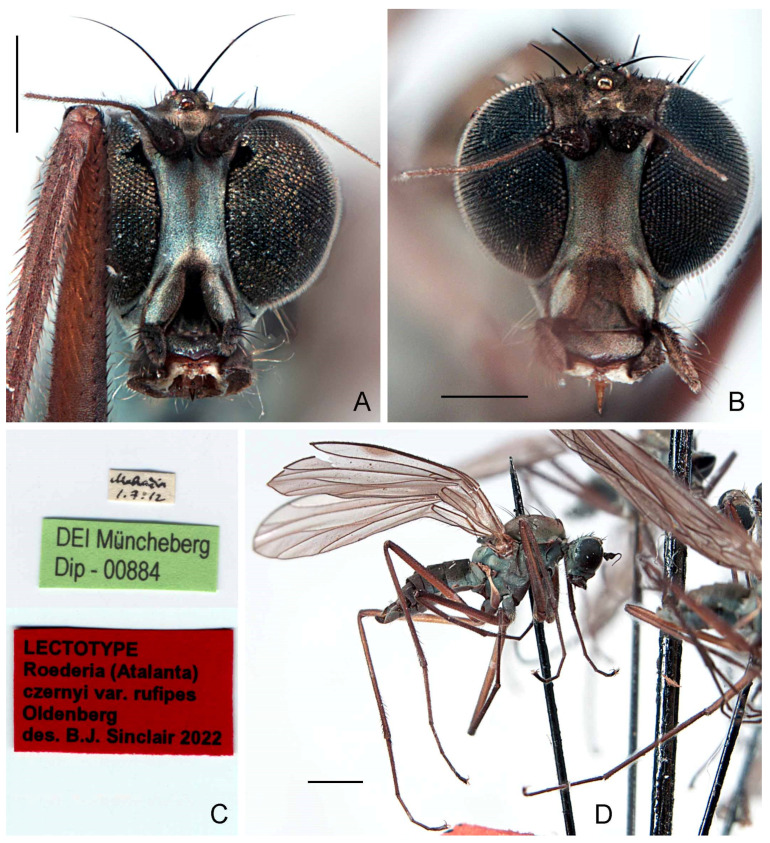
*Wiedemannia rufipes* (Oldenberg). (**A**) male head, frontal view, scale bar = 0.25 mm; (**B**) female head, frontal view, scale bar = 0.25 mm; (**C**) lectotype labels; (**D**) lectotype habitus, scale bar = 1.0 mm.

1. Face brown similar to frons (Figure 10A), concolourous with scutum, contrasting with blue pruinescent gena; wing with stout erect setae on costa starting near humeral crossvein (Figure 10C); clasping cercus same colour as epandrium, bilobed, lobes not in same plane, with prominent anterior lobe projecting obliquely from posterior lobe (Figure 10B), anterior lobe usually distinctly longer than posterior lobe (Figure 3H); wings usually shorter than 5 mm ……………….. ***W. zetterstedti*** (Fallén)

- Face with blue pruinescence (Figure 2A, Figure 4A and Figure 5A, if brown (female *W. rufipes*), then with narrow, dark median stripe (Figure 5B); frons brown; wing with stout erect setae on costa starting beyond apex of Sc; clasping cercus colour usually distinctly paler than epandrium (Figure 2B and Figure 4B); anterior and posterior lobes of clasping cercus in same plane; anterior lobe not distinctly longer than posterior lobe (Figure 3A–F); wing often longer than 5 mm……………………………………………………. 2

2. Hind tibia with at least 4–5 erect spine-like posterodorsal setae apically (Figure 4E); clasping cercus narrow (Figure 4B), with apical lobes similar in width, at most posterior lobe slightly broader than anterior lobe; thickened setae on inner face confined to posterior lobe (Figure 3C)……………………………………… ***W. longipennis*** (Mik)

- Hind tibia without or with 2–3 erect spine-like posterodorsal setae apically (Figure 2D and Figure 7D); clasping cercus broad, mitten-shaped (Figure 3A); posterior lobe broader, more than twice width of anterior lobe; thickened setae on inner face extended basally beyond posterior lobe (Figure 3B)……………………………………... 3

3. Hind tibia without erect, spine-like posterodorsal setae apically (Figure 7D)………………………………………………………………………. ***W. ulrichi*** sp. nov.

- Hind tibia with 2–3 erect, spine-like posterodorsal setae apically (Figure 2D)……… 4

4. Male face with bluish pruinescence and dark central stripe (Figure 5A); female face brownish blue with faint central stripe (Figure 5B)…………... ***W. rufipes*** (Oldenberg)

- Face of male and female with bluish pruinescence, without dark central stripe (Figure 2A)………………………………………………………………………. ***W. czernyi***(Bezzi)

#### 3.2.3. *Wiedemannia czernyi* (Bezzi)

(Figure 2A–E, Figure 3A,B and Figure 11)
*Clinocera* (*Roederia*) *czernyi* Bezzi, 1905: 362. Type locality (by lectotype designation): Acquasanta, Macerata Province, Italy.
*Atalanta* (*Roederella*) *czernyi*: Engel 1918: 197; Engel 1940: 162.
*Wiedemannia* (*Roederella*) *czernyi*: Melander 1928: 242, 244.
*Wiedemannia* (*Roederella*) *ouedorum* Vaillant, 1952: 371. Type locality: Djurdjura and Aurès mountains, Algeria. syn. nov.
*Wiedemannia* (*Roederella*) *ouedorum*: Ivković et al. 2014a: 553; Ivković et al. 2019: 563.

**Type material examined.***Clinocera czernyi*: **LECTOTYPE** (here designated in order to fix identity of the species) ♂, labelled (Figure 2C): “Acquasant. [Aquasanta Gorge, 1205 m, Sibillini Mountains near Bolognola, Macerata Province, Marche Region, Central Italy, 42°58′46″ N 13°11′49″ E]/3.VIII.97”; “Museo/Storia Naturale/MILANO”; “LECTOTYPE/*Clinocera* (*Roederia*)/*czernyi* Bezzi/des. B.J. Sinclair 2022 [red label]” (MSNM). **PARALECTOTYPES:** same data as lectotype [dissected] (1 ♂, MSNM); same data as lectotype except, 4.viii.1897 (1 ♂, 1 ♀, USNM); same data as lectotype except, 9.viii.1897 (1 ♂, 1 ♀, USNM).

**Notes on type material.** Bezzi [21] stated that this species was collected in the central Italian mountains, from specimens initially identified as *Roederia longipennis* Mik in Bezzi [22]. In the latter publication, numerous specimens were collected along the streams “Fiastone, Acquasanta, Tennacola and Tronto above Aquata” in Macerata Province, Marche Region, Central Italy. Upon review of specimens received from Czerny that were reportedly *R. longipennis*, Bezzi [21] reconsidered his identification and described them as a new species. But in an extract of a letter to Engel, Czerny clarifies that he sent Bezzi specimens of *Eucelidia escheri* Zetterstedt [9] (p. 755), [23] (p. 79). This observation led Engel [23] to assume *R. longipennis* was conspecific with *E*. *escheri*, and consequently he synonymized the former with the latter, assigned *Roederia* in synonymy with *Eucelidia* and described a new genus, *Roederella* for *C. czernyi*. Collin [9] examined the types of *Roederia longipennis* and considered them conspecific with *Roederella czernyi* and that the description of the new genus was unjustified.

Unfortunately, the definition of *W. czernyi* is somewhat more complicated than outlined by Collin [9]. More than 20 syntypes have been identified in MSNM (Rigato 2020, pers. comm.), and we received six purported syntypes, representing a mixed series. Two syntypes represent *W. czernyi* in its current concept and three conspecific with *W. longipennis* based on comparison with type specimens of the latter species (sixth specimen was *W. longicornis* (Mik)). Interestingly, the illustration of the male terminalia of *W. czernyi* [21] (Figure 1) appears very similar to *W. longipennis*. Doubts on the definition of the species was further compounded by Engel [23] (Figures 26 and 27), who illustrated a dried and macerated specimen of “*W. czernyi*”, but they appear to represent two different species, with the dried specimen (Figure 26) matching the types of *W. longipennis* and the macerated specimen (Figure 27) matching *W. czernyi*. Rather than synonymize these two species and describe a new species, we have chosen to maintain nomenclatural stability by selecting a lectotype specimen from the two representatives that most closely match the current concept of *W. czernyi*.

The private collection of F. Vaillant is now located in Musée cantonal de zoologie, Lausanne, Switzerland [24]. Unfortunately, only larval syntypes of *W. ouedorum* could be found and adults are presumed destroyed (Gattolliat pers. comm. 2020). The proposed new synonym is based on the illustrations by Vaillant [25] (Figures 25–29) compared to specimens from Spain, as well as a non-type specimen determined by F. Vaillant from Algeria (ZISP).

**Additional material examined. Algeria:** Lac Goulmine (36°27′52″ N 04°4′1″ E, 1756 m), 18.vi.1953, F. Vaillant coll. (1 ♂, ZISP). **Italy:** Abruzzen (ca 42.284° N 13.730° E), 21.v.1977, leg. Zwick (1 ♂, Ivković coll. UZC; 3 ♂, CNC); Vallombrosa (43.732° N 11.551° E), 10.vi., 16.vi.1908, Oldenberg (5 ♂, 3 ♀, USNM). **Spain:** Sierra Nevada, Río Genil, La Fabriquilla, 37°09′21″ N 03°26′00″ E, 960 m, 13.v.2013, M. Ivković (3 ♂, 1 ♀, CNC); Rio Guadarrama, 3.vi.1961, 1200 m, Inst. Esp. Ent., Mhs (B. Mannheims) (1 ♂, 5 ♀, ZFMK).

**Diagnosis.** This species is distinguished from other species of the *Wiedemannia zetterstedti* group by the following combination of characters: face bluish pruinescent in both sexes, without brownish stripe; wing often longer than 5 mm; stout erect setae on costa beyond vein Sc; hind tibia with at least 2–3 erect spine-like posterodorsal setae apically; clasping cercus pale brown, mitten-shaped.

**Redescription.** Wing length 4.7–5.2 mm. **Male** (Figure 2E). Head with gena, postgena and lower half of occiput with blue-grey pruinescence; gena less than half as deep as eye height. Face slightly wider than antennal sockets, with bluish pruinescence (Figure 2A); distinct carina on lower margin. Frons short, brown, broader than face; ocellar triangle and vertex brown. Pair of long ocellar setae; one pair of strong vertical setae; 5–6 distinct upper postocular setae, all similarly sized and black, with interspersed shorter setae; lower postocular setae much finer and paler, merging with pale, longer setae on middle and lower occiput and postgena; several dark setulae on vertex and between ocellar area and eye margin. Antenna brown; scape and pedicel subequal; scape with dorsal setulae; pedicel with circlet of apical setulae; postpedicel pointed ovate; arista-like stylus shorter than length of face.

Antepronotum with pair of lateral setae, one of which nearly same length as postocular setae. Proepisternum with some long, fine pale setae. Scutum brown, faintly bivittate; scutellum concolourous; postpronotal lobe and lower margin of notopleuron sometimes with faint blue-grey pruinescence. Pleura with bright bluish pruinescence. Mesonotum with 5 pairs of dorsocentral setae; several short acrostichal setulae, irregularly biserial, anterior to second dorsocentral seta; 1 postpronotal seta, with several short, dark setulae; 2 notopleural setae, usually with dark setulae on lower half; 1 presutural supra-alar seta, usually with several dark setulae; 0 postsutural supra-alar setae; 1 postalar seta; 1 pair scutellar setae with dark marginal and discal setulae. Laterotergite with cluster of pale setae.

Wing membrane infuscate, veins dark; basal costa seta not extending to humeral crossvein. Origin of veins M_1_ and M_2_ widely separated at end of cell dm. Vein CuA + CuP short, faint streak, or absent. Pterostigma narrow, elongate, faint to strong. Squama with pale fringes. Costal margin with short, strong erect setae beyond apex of Sc. Halter with dark knob and paler shaft.

Legs entirely dark brown, except coxae with pale grey pruinescence; uniformly covered with rows of small dark setulae, slightly longer on ventral side of fore femur; fore femur often with strong anterior preapical seta; fore coxa with 2–3 long erect setae; hind tibia with 2–3 erect, spine-like posterodorsal setae (Figure 2D).

Abdomen concolourous with thorax, covered in short setae; lateral third of tergites and sternites with bluish pruinescence. Terminalia (Figure 2B and Figure 3A,B): Hypandrium slightly shorter than length of epandrium, bearing 2−3 setae with bluish pruinescence. Epandrium elongate, oval, covered with dark, long setae; rounded surstylus emerging from inner face apically. Clasping cercus pale brown, paler than epandrium; shorter than length of epandrium; bifurcated (bilobed), mitten-shaped with lobes in same plane; anterior lobe much narrower than broad posterior lobe, with long outer setae; posterior lobe with inner face with long, peg-like setae; anterior and posterior lobes variable in size. Phallus more or less linear, brownish; distiphallus straight, without swelling in middle.

**Female.** Similar to male except terminalia; cercus short, cylindrical, and minutely pilose.

**Distribution.** This species is confirmed from Algeria, France, Italy, Morocco, and Spain (Figure 11).

**Remarks.**  *The size of the apical lobes of the clasping cercus is variable in size, confusing species identification (Figure 2B and Figure 3A). Additional features outlined in the key are needed to aid species identification*.

#### 3.2.4. *Wiedemannia longipennis* (Mik) stat. rev.

(Figure 3C,D, Figure 4A–F, and Figure 11)
*Clinocera longipennis* Mik, 1880; 349. Type locality: Romania.
*Roederia longipennis*: Mik 1881: 326.
*Eucelidia longipennis*: Engel 1918: 202 (as synonym of *E. escheri*).
*Wiedemannia* (*Eucelidia*) *longipennis*: Melander 1928: 243 (as synonym of *W. escheri*).

**Type material examined. LECTOTYPE** (here designated in order to fix identity of the species) ♂ [dissected; Figure 4C], labelled (Figure 4D): “SYNTYPE”; “H??ňlbd [illegible, probably Herkulesbad]/5.6.[1]871”; “Typ Mik” “*Röderia*/*longipennis*/ Mik ♂”; “*C./longipennis*/Mik/F. Kow.”; “*C. longipennis*/ex COLL. KOW.”; “LECTOTYPE/*Clinocera longipennis*/Mik/des. M. Ivković 2019 [red label]” (OUMNH). **PARALECTOTYPE:** same data as lectotype, except 1.6.1871 [dissected] (1 ♂, OUMNH).

**Notes on type specimens.** This species was described on the basis of an undetermined number of male and female specimens, and Mik [26] stated that the specimens were from “Hungary” (Herkulesbad was in the Austro-Hungarian Empire, now Romania), presented to him by F. Kowarz. Mik [27] transferred the species to his new genus *Roederia*. Engel [23] (p. 79) wrote that the types in the “Wiener Hofmuseum” were either destroyed or in poor shrunken condition. Additional type specimens identified in the Hope Collection were from the Kowarz collection, which was purchased by Verrall in the 1890s [28], also mentioned in Collin [9] (p. 755). See additional comments under *W. czernyi*.

**Additional material examined. Croatia:** Dalmatia, Kosovčica Spring, 31.viii.2011, 260 m, 43°56′28″ N 16°15′10″ E, M. Ivković (5 ♂, CNC); same data except, 14.x.2011 (3 ♂, 1 ♀, CNC); Spring Krčič, 18.xi.2010, 390 m, 44°01′48″ N 16°19′42″ E, M. Ivković (3 ♂, 2 ♀, CNC). **Italy:** Acquasanta, 13.viii.1893 (1 ♀, paralectotype *C. czernyi*, MSNM), same data except, 4.viii.1897 (1 ♂, paralectotype *C. czernyi*, USNM); Farnio (=Fargno, Sibillini Mountains near Bolognola, Macerata Province, Marche Region, Central Italy, 42°57′12″ N 13°12′29″ E), 27.vi.1897 (1 ♂, paralectotype *C. czernyi*, MSNM); Tennacola (Macerata Province, Marche Region, Central Italy, 42°59′22″ N 13°15′22″ E), 19.vi.1897 (1 ♂, paralectotype *C. czernyi*, MSNM); Vallombrosa (43.732° N 11.551° E), 10.vi., 16.vi.1908, Oldenberg (2 ♀, USNM). **Lebanon:** St. 7, Riv. Awali, Nabaa Mourched (Ouâdi Qâchqich), près du village d’El Moukhtâra, 800 m, 8.v.1981, A. Dia (2 ♂ GBIFCH00602226, MZLS); St. 13, Nabaa Aazibi, affluent de l’ Awali, 24.v.1980, A. Dia (2 ♂ GBIFCH00602228, 1 ♂ GBIFCH00602230, MZLS); St. 25, Riv. Damour, en aval de la confluence de Nabaa es Safa et ruisseau Aîn Dara, 22.viii.1980, A. Dia (1 ♂ GBIFCH00602227, 2 ♂, 1 ♀ GBIFCH00602229, MZLS); St. 1, Riv. Awali, Nabaa el Barouk, près du village d’El Barouk, 8.iv.1984, A. Dia (1 ♂ GBIFCH00602232, MZLS). **Montenegro:** Alipaša’s Springs, 6.vii.2012, M. Ivković (4 ♂, CNC).

**Diagnosis.** This species is distinguished from other species of the *Wiedemannia zetterstedti* group by the following combination of characters: face bluish pruinescent in both sexes, without brownish stripe; wing usually longer than 5 mm; stout erect setae on costa beyond vein Sc; hind tibia with at least 4–5 erect, spine-like posterodorsal setae apically; clasping cercus pale brown, narrow, with posterior lobe slightly broader than anterior lobe, thickened setae on inner face confined to posterior lobe.

**Redescription.** Wing length 5–5.5 mm. **Male** (Figure 4F). Head with gena, postgena and lower half of occiput with blue-grey pruinescence; gena less than half as deep as eye height. Face slightly wider than antennal sockets, with bluish pruinescence (Figure 4A); distinct carina on lower margin. Frons short, brown, broader than face; ocellar triangle and vertex brown. Pair of long ocellar setae; one pair of strong vertical setae; 5–6 distinct upper postocular setae, all similarly sized and black, with interspersed shorter setae; lower postocular setae much finer and paler, merging with pale, longer setae on middle and lower occiput and postgena; several dark setulae on vertex and between ocellar area and eye margin. Antenna brown; scape and pedicel subequal; scape with dorsal setulae; pedicel with circlet of apical setulae; postpedicel pointed ovate; arista-like stylus shorter than length of face.

Antepronotum with row of short setae, 1–2 slightly stronger. Proepisternum with some long, fine pale setae. Scutum brown, faintly bivittate; scutellum concolourous; postpronotal lobe and lower margin of notopleuron sometimes with faint blue-grey pruinescence. Pleura with bright bluish pruinescence. Mesonotum with 5 pairs of dorsocentral setae; several short acrostichal setulae, irregularly biserial, anterior to second dorsocentral seta; 1 postpronotal seta, with several short, dark setulae; 2 notopleural setae, usually with dark setulae on lower half; 1 presutural supra-alar seta, usually with several dark setulae; 0 postsutural supra-alar setae; 1 postalar seta; 1 pair scutellar setae with dark marginal and discal setulae. Laterotergite with cluster of pale setae.

Wing membrane infuscate, veins dark; basal costa seta not extending to humeral crossvein. Origin of veins M_1_ and M_2_ widely separated at end of cell dm. Vein CuA + CuP short, faint streak or absent. Pterostigma narrow, elongate, faint to strong. Squama with pale fringes. Costal margin with short, strong erect setae beyond apex of Sc. Halter with dark knob and paler shaft.

Legs entirely dark brown, except coxae with pale grey pruinescence; uniformly covered with rows of small dark setulae, slightly longer on ventral side of fore femur; fore femur often with strong anterior preapical seta; fore coxa with 2–3 long erect setae; hind tibia with 4–10 erect, spine-like posterodorsal setae (Figure 4E).

Abdomen concolourous with thorax, covered in short setae; lateral third of tergites and sternites with bluish pruinescence. Terminalia (Figure 3C,D and Figure 4B): Hypandrium nearly subequal in length with epandrium, bearing 1−2 setae with bluish pruinescence. Epandrium elongate, oval, covered with dark, long setae; rounded surstylus emerging from inner face apically. Clasping cercus pale brown, paler than epandrium; long and narrow, subequal to length of epandrium; bifurcated (bilobed), with lobes in same plane and posterior lobe slightly broader than anterior lobe; anterior lobe flatter than posterior lobe, with short outer setae; longer setae in area separating lobes; posterior lobe thumb-like with short setae along margin; inner face with peg-like setae confined distinctly to posterior lobe. Phallus more or less linear, brownish; distiphallus straight, without swelling in middle.

**Female.** Similar to male except terminalia; cercus short, cylindrical, and minutely pilose.

**Distribution.** This species is recorded from Bosnia and Herzegovina, Bulgaria, Croatia, Georgia, Greece (incl. Aegean Islands), Italy, Lebanon, Cyprus, Montenegro, North Macedonia, Romania, Serbia, Slovenia, and Turkey (Figure 11).

**Remarks.**  *This species was synonymized with W. escheri (Zetterstedt) by Engel [23] (p. 80), and its concept remained as such until we re-examined the type specimens of W. longipennis. The long and narrow clasping cercus and numerous posterodorsal setae of the hind tibia readily distinguishes W. longipennis from all other species of the W. zetterstedti group*.

#### 3.2.5. *Wiedemannia rufipes* (Oldenberg) stat. rev.

**Figure 6:**
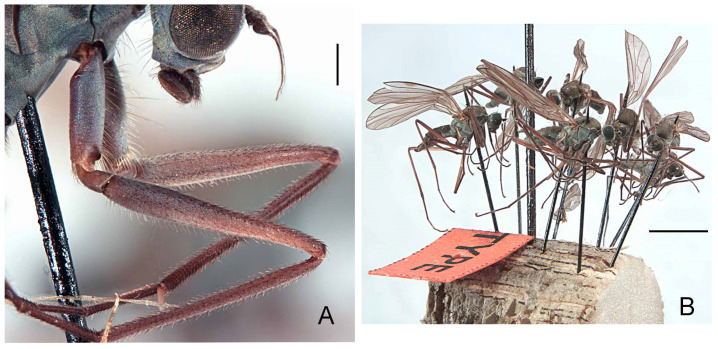
*Wiedemannia rufipes* (Oldenberg). (**A**) head and fore legs, lateral view, scale bar = 0.25 mm; (**B**) type specimens, scale bar = 2.5 mm.

(Figure 3E, Figure 5A–D, Figure 6A,B and Figure 11)
*Roederia* (*Atalanta*) *czernyi* var. *rufipes* Oldenberg, 1915: 92. Type locality: “Seitenbach der Cserna eine Stunde oberhalb Herkulesbad” (Băile Herculane, Romania).
*Atalanta* (*Roederella*) *czernyi* var. rufipes: Engel 1918: 198.
*Wiedemannia* (*Roederella*) *czernyi* var. rufipes: Melander 1928: 244.
*Atalanta* (*Roederella*) *czernyi* rufipes: Engel 1940: 162.

**Type material examined. LECTOTYPE** (here designated in order to fix identity of the species) ♂ (Figure 5D and Figure 6B), labelled (Figure 5C): “Mehadia [ca 44.875, 22.401]/1.7.1912”; “Coll./Oldenberg”; “LECTOTYPE/*Clinocera* (*Roederia*)/*czernyi* Bezzi/des. B.J. Sinclair 2022 [red label]” (1 of 8 specimens mounted on cork; small red type label attached next to pin) (SDEI). **PARALECTOTYPES:** Same data as lectotype (5 ♂, 12 ♀, SDEI); Mehadia, 26.vi.[19]12, Oldenberg (2 ♂, MSNM).

**Notes on type material.** Oldenberg [29] described the type locality of *W. rufipes* as “Seitenbach der Cserna eine Stunde oberhalb Herkulesbad” (side stream of the Cserna one hour above Hercules Bath). The 18 syntypes found in SDEI are all labelled “Mehadia”, a town on the river Belareca, which joins the river Cserna downstream from Băile Herculane (Romania). It is likely that Oldenberg used the name Mehadia to describe this region where the rivers meet.

**Additional material examined. Georgia:** Samagrelo, Oftizari ridge, stream, N42°35′28.7″, E42°22′36.1″, 828 m, 6.v.2014, Zherebilo D.A. leg. (1 ♂, Ivković coll. UZC). **Greece:** Macedonia, CHA, Chalkidiki, Chlomon Oros., Paleokastron, Vatonia P., QN 2522, 500 m, 28.v.1991, R. Gerecke (1 ♂, CNC); Thrace, E Sapka Mts, 600 m, N41°08′00″, E52°57′00″, 30.v.1989, big stream in valley, H. Malicky (1 ♂, CNC).

**Diagnosis.** This species is distinguished from other species of the *Wiedemannia zetterstedti* group by the following combination of characters: male face bluish pruinescent or female face brownish-blue, both with a brownish median stripe; wing often longer than 5 mm; stout erect setae on costa beyond vein Sc; legs often reddish brown; hind tibia with at least 2–3 erect spine-like posterodorsal setae apically; clasping cercus pale brown, mitten-shaped.

**Redescription.** Wing length 4.8–5.8 mm. **Male.** Head with gena, postgena and lower half of occiput with blue-grey pruinescence; gena less than half as deep as eye height. Face slightly wider than antennal sockets, with bluish pruinescence and brown, narrow median stripe (Figure 5A); distinct carina on lower margin. Frons short, brown, broader than face; ocellar triangle and vertex brown. Pair of long ocellar setae; one pair of strong vertical setae; 5–6 distinct upper postocular setae, all similarly sized and black, with interspersed shorter setae; lower postocular setae much finer and paler, merging with pale, longer setae on middle and lower occiput and postgena; several dark setulae on vertex and between ocellar area and eye margin. Antenna brown; scape and pedicel subequal; scape with dorsal setulae; pedicel with circlet of apical setulae; postpedicel pointed ovate; arista-like stylus nearly subequal to length of face.

Antepronotum with several lateral setae, one of which nearly length of postocular setae. Proepisternum with some long, fine pale setae. Scutum brown, faintly bivittate; scutellum concolourous; postpronotal lobe and lower margin of notopleuron sometimes with faint blue-grey pruinescence. Pleura with bright bluish pruinescence. Mesonotum with 5 pairs of dorsocentral setae; several short acrostichal setulae, irregularly biserial, anterior to second dorsocentral seta; 1 postpronotal seta; 2 notopleural setae, usually with dark setulae on lower half; 1 presutural supra-alar seta, usually with several dark setulae; 0 postsutural supra-alar setae; 1 postalar seta; 1 pair scutellar setae with dark marginal and discal setulae. Laterotergite with cluster of pale setae.

Wing membrane infuscate, veins dark; basal costa seta extending to humeral crossvein. Origin of veins M_1_ and M_2_ widely separated at end of cell dm. Vein CuA+CuP short, faint streak or absent. Pterostigma narrow, elongate, faint to strong. Squama with pale fringes. Costal margin with short, strong erect setae beyond apex of Sc. Halter with dark knob and paler shaft.

Legs often reddish brown, especially on ventral surface (Figure 6A); coxae with pale grey pruinescence; uniformly covered with rows of small dark setulae, slightly longer on ventral side of fore femur; fore femur often with strong anterior preapical seta; fore coxa with 2–3 long erect setae; hind tibia with 2–3 erect, spine-like posterodorsal setae.

Abdomen concolourous with thorax, covered in short setae; lateral third of tergites and sternites with bluish pruinescence. Terminalia (Figure 3E): Hypandrium slightly shorter than length of epandrium, bearing 1−2 setae with bluish pruinescence. Epandrium elongate, oval, covered with dark, long setae; rounded surstylus emerging from inner face apically. Clasping cercus pale brown (difficult to discern in air-dried older specimens)? Paler than epandrium; shorter than length of epandrium; bifurcated (bilobed), mitten-shaped with lobes in same plane; anterior lobe much narrower than broad posterior lobe, with long outer setae; posterior lobe with long setae along outer posterior margin; inner face of posterior lobe with long, peg-like setae. Phallus more or less linear, brownish; distiphallus straight, without swelling in middle.

**Female.** Similar to male except, face brownish blue with faint median strip (Figure 5B); terminalia with cercus short, cylindrical, and minutely pilose.

**Distribution.** This species is recorded from Georgia, Greece, and Romania (Figure 11).

**Remarks.**  *This species was originally considered a subspecies of W. czernyi, distinguished from the latter species by reddish-coloured legs [29]. Leg colouration is often not a dependable character to distinguish species and can by modified during preservation of specimens. The features in the key help to further characterize this species*.

#### 3.2.6. *Wiedemannia ulrichi* Ivković & Sinclair sp. nov.

**Figure 7:**
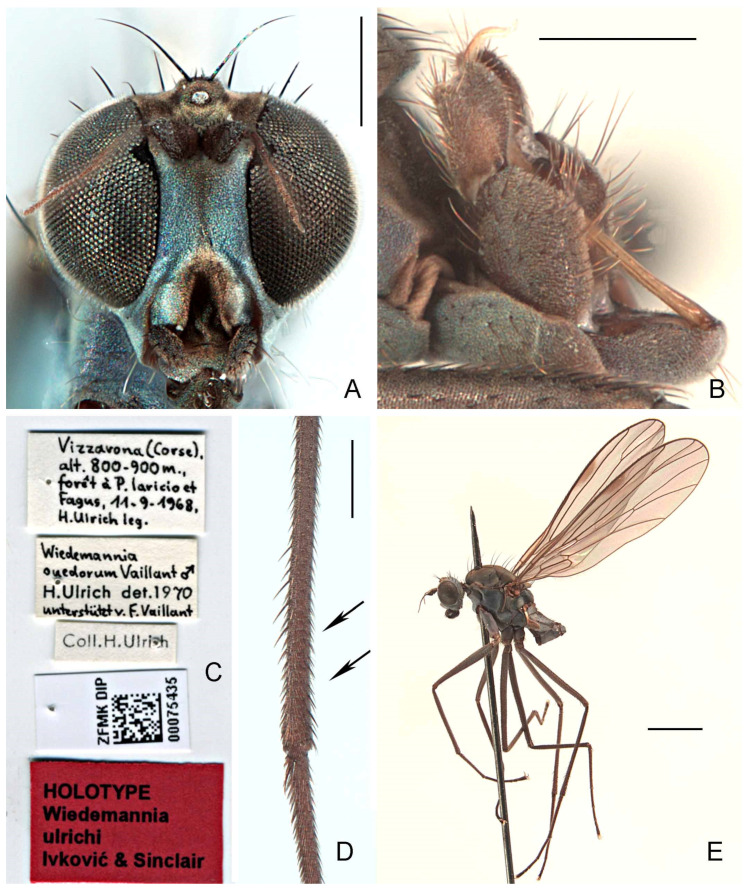
*Wiedemannia ulrichi* sp. nov. (**A**) head, frontal view, scale bar = 0.25 mm; (**B**) male terminalia, oblique lateral view, scale bar = 0.25 mm; (**C**) holotype labels; (**D**) hind tibia, posterior view, arrows showing that there is no spine-like posterodorsal setae, scale bar = 0.25 mm; (**E**) paratype habitus (dissected), lateral view, scale bar = 1.0 mm.

(Figure 3F, Figure 7A–E and Figure 11)
urn:lsid:zoobank:org:act:312BA60E-97B0-495C-83CB-7C81078BF269

**Type material. HOLOTYPE** ♂, labelled (Figure 7C): “Vizzavona (Corse),/alt. 800–900 m.,/forêt à P. laricio et/Fagus, 11-9[ix]-1968,/H. Ulrich leg.”; *Wiedemannia*/*ouedorum* Vaillant ♂/H. Ulrich det. 1970/unterstützt v. F. Vaillant”; “Coll. H. Ulrich”; “ZFMK DIP/[data matrix code]/ 0075435”; “HOLOTYPE/*Wiedemannia*/*ulrichi*/Ivković & Sinclair [red label]” (ZFMK). **PARATYPES: France:** Corsica: Zicavo, Ponte di Valpine, 25.vi.2019, 1264 m, N41°52′29.0″ E09°08′04.7″, waterfall in riverbed, M. Pollet (1 ♂, 2 ♀, Ivković coll. UZC); same data except, 29.vi.2019, 1277 m, N41°52′27.6″ E09°08′06.8″, dry rocks, seeps in riverbed (1 ♂, 3 ♀, MNHM); same data as holotype (1 ♂, 1 ♀, CNC; 3 ♂, 4 ♀, ZFMK).

**Diagnosis.** This species is distinguished from other species of the *Wiedemannia zetterstedti* group by the following combination of characters: face blue pruinescent in both sexes, with complete central brownish stripe; wing length variable, sometimes longer than 5 mm; stout erect setae on costa beyond vein Sc; hind tibia without distinct erect spine-like posterodorsal setae apically; clasping cercus pale brown, mitten-shaped, shallowly notched with posterior lobe broader than anterior lobe.

**Description.** Wing length 4.6–5.5 mm. **Male** (Figure 7E). Head with gena, postgena, and lower half of occiput with blue-grey pruinescence; gena less than half as deep as eye height. Face slightly wider than antennal sockets, with bluish pruinescence and brown median stripe (Figure 7A); distinct carina on lower margin. Frons short, brown, broader than face; ocellar triangle and vertex brown. Pair of long ocellar setae; one pair of strong vertical setae; 5–6 distinct upper postocular setae, all similarly sized and black, with interspersed shorter setae; lower postocular setae much finer and paler, merging with pale, longer setae on middle and lower occiput and postgena; several dark setulae on vertex and between ocellar area and eye margin. Antenna brown; scape and pedicel subequal; scape with dorsal setulae; pedicel with circlet of apical setulae; postpedicel pointed ovate; arista-like stylus shorter than length of face.

Antepronotum with pair of lateral setae, one of which strong, nearly length of postocular setae. Proepisternum with some long, fine pale setae. Scutum brown, faintly bivittate; scutellum concolourous; postpronotal lobe and lower margin of notopleuron sometimes with faint blue-grey pruinescence. Pleura with bright bluish pruinescence. Mesonotum with 5 pairs of dorsocentral setae; several short acrostichal setulae, irregularly biserial, anterior to second dorsocentral seta; 1 postpronotal seta; 2 notopleural setae, usually with dark setulae on lower half; 1 presutural supra-alar seta, usually with several dark setulae; 0 postsutural supra-alar setae; 1 postalar seta; 1 pair scutellar setae with dark marginal and discal setulae. Laterotergite with cluster of pale setae.

Wing membrane infuscate, veins dark; basal costa seta extending to humeral crossvein. Origin of veins M_1_ and M_2_ widely separated at end of cell dm. Vein CuA + CuP short, faint streak or absent. Pterostigma narrow, elongate, faint to strong. Squama with pale fringes. Costal margin with short, strong erect setae beyond apex of Sc. Halter with dark knob and paler shaft.

Legs entirely dark brown, except coxae with pale grey pruinescence; uniformly covered with rows of small dark setulae, slightly longer on ventral side of fore femur; fore femur often with strong anterior preapical seta; fore coxa with 2–3 long erect setae; hind tibia without erect, spine-like posterodorsal setae (Figure 7D).

Abdomen concolourous with thorax, covered in short setae; lateral third of tergites and sternites with bluish pruinescence. Terminalia (Figure 3F and Figure 7B): Hypandrium slightly shorter than length of epandrium, bearing 1−2 setae with bluish pruinescence. Epandrium elongate, oval, covered with dark, long setae; rounded surstylus emerging from inner face apically. Clasping cercus pale brown, paler than epandrium; shorter than length of epandrium; bifurcated (bilobed), mitten-shaped with lobes in same plane, shallowly separated; anterior lobe much narrower than broad posterior lobe, with long outer marginal setae; posterior lobe without long outer setae; inner face of posterior lobe with long, peg-like setae. Phallus more or less linear, brownish; distiphallus straight, without swelling in middle.

**Female.** Similar to male except terminalia; cercus short, cylindrical, and minutely pilose.

**Distribution.** This species appears to be endemic to Corsica (France) (Figure 11).

**Remarks.**  *In a study of the aquatic Empididae of Corsica, Pusch [30] and Ivković et al. [31] identified this new species as Wiedemannia czernyi. Wiedemannia ulrichi sp. nov. is the most frequently encountered species of *Clinocerinae*, collected between 80 and 1700 m and found in sunny or shady sections of narrow to broad rivers [30]*.

Additional examples of the endemic *Clinocerinae* fauna from Corsica include: *Clinocerella wagneri* (Pusch), *Kowarzia cataractae* (Pusch), *K. schnabli* Becker, *Wiedemannia ariolae* Pusch, *W. bravonae* Pusch, *W. corsicana* Vaillant, and *W. martini* Pusch. For additional information on the endemic aquatic Empididae from Corsica, see Ivković et al. [31].

#### 3.2.7. *Wiedemannia zetterstedti* (Fallén)

**Figure 8:**
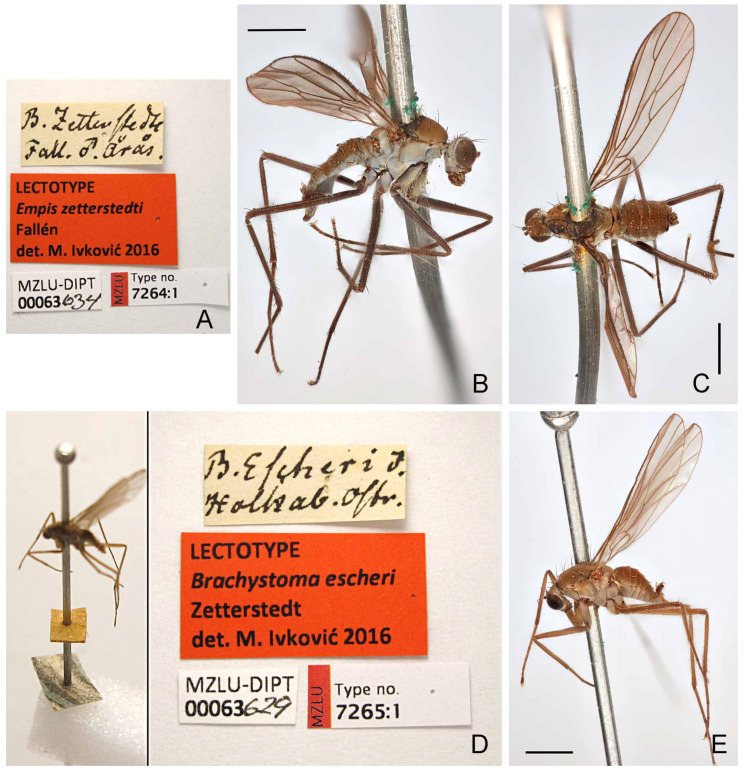
*Wiedemannia* lectotype specimens and labels. (**A**) *W. zetterstedti* (Fallén), labels; (**B**) *W. zetterstedti*, habitus, lateral view; (**C**) *W. zetterstedti*, habitus, dorsal view; (**D**) *W. escheri* (Zetterstedt), habitus and labels; (**E**) *W. escheri*, habitus, oblique lateral view. Scale bars = 1 mm. All images courtesy of the Lund Museum of Zoology, Lund University (MZLU).

**Figure 9:**
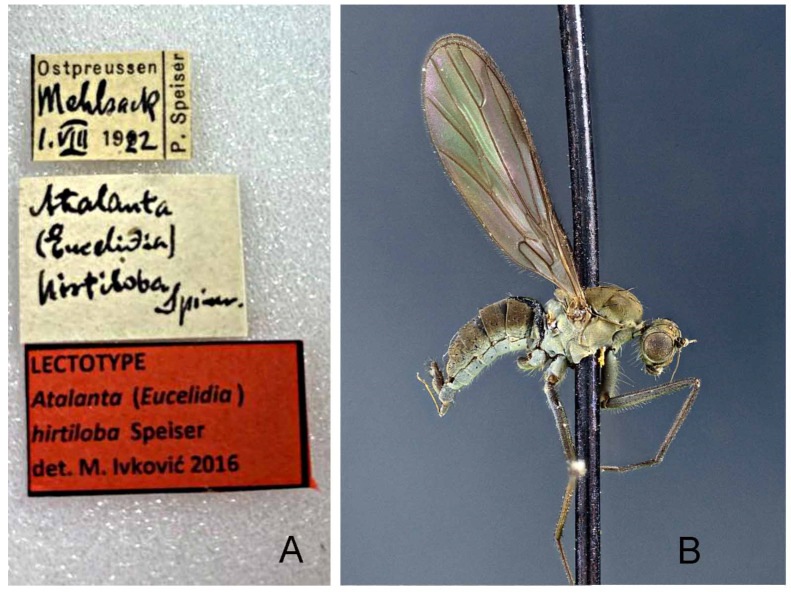
*Wiedemannia hirtiloba* (Speiser). (**A**) lectotype labels; (**B**) lectotype habitus. Images courtesy Naturhistorisches Museum, Wien (NMW).

**Figure 10:**
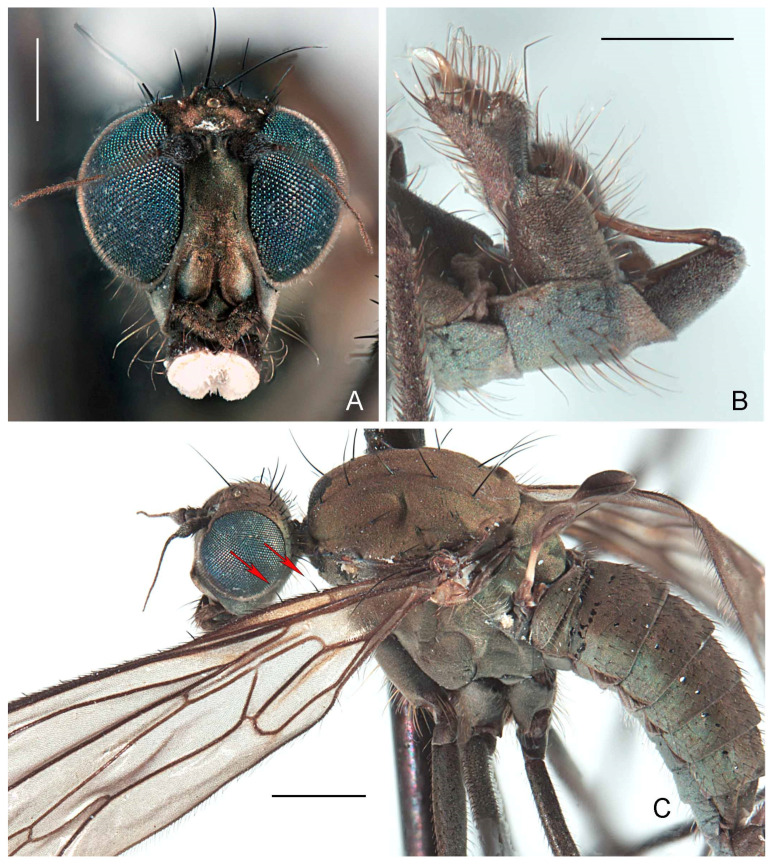
*Wiedemannia zetterstedti* (Fallén). (**A**) Head, frontal view, scale bar = 0.25 mm; (**B**) male terminalia, lateral view, scale bar = 0.25 mm; (**C**) habitus, dorsolateral view, arrows pointing to erect costal setae, scale bar = 0.5 mm.

**Figure 11:**
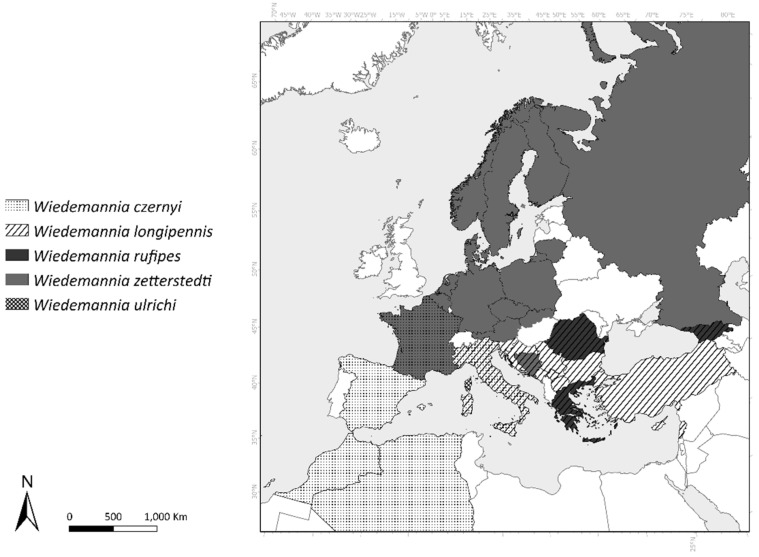
Distribution of all species of the *W. zetterstedti* group by country.

(Figure 3G,H, Figure 8A–E, Figure 9A,B, Figure 10A–C and Figure 11)
*Empis zetterstedti* Fallén, 1826: 7. Type locality: “Årås Vermlandiae juxta lacum Vänern” (Årås by lake Vänern, Värmland, Sweden).
*Paramesia riparia* Robert, 1836: 538 [32]. Type locality: Liege, Belgium. syn. nov.
*Brachystoma escheri* Zetterstedt, 1838: 558. Type locality: Holkaberg, Östergötland, Sweden [58.123141, 14.557056] (by lectotype designation).
*Clinocera zetterstedti*: Loew 1858: 249 [33].
*Eucelidia zetterstedti*: Mik 1881: 327; Engel 1918: 203.
*Atalanta* (*Eucelidia*) *zetterstedti*: Engel 1918: 203; Engel 1940: 161.
*Atalanta* (*Eucelidia*) *hirtiloba* Speiser, 1924: 13. Type locality: “Walsehtal, Ostpreussen” (= near Pieniężno, Warmia-Masuria, Poland).
*Wiedemannia* (*Eucelidia*) *zetterstedti*: Melander 1928: 241, 243.

**Type material examined.***Empis zetterstedti* Fallén: **LECTOTYPE** (here designated in order to fix identity of the species) ♂ (Figure 8B,C), labelled (Figure 8A): “*B. zetterstedti*/ Fall. ♂ Årås.”; “LECTOTYPE/*Empis zetterstedti*/Fallén/det. M. Ivković 2016”; “MZLU-DIPT/00063634”; “MZLU/Type no./7264:1” (MZLU). **PARALECTOTYPES:** Årås (1♂, 2♀, MZLU).

*Brachystoma escheri* Zetterstedt: **LECTOTYPE** (here designated in order to fix identity of the species) ♂ (Figure 8E), labelled (Figure 8D): “[yellow square]/[bicolored blue/white square]/*B. escheri* ♂/Holkab. Ostr.”; “LECTOTYPE/*Brachystoma escheri*/Zetterstedt/det. M. Ivković 2016”; “MZLU-DIPT/00063629”; “MZLU/Type no./7265:1” (MZLU). **PARALECTOTYPES:** same data as lectotype (1 ♀, MZLU); var. b ♂ m, Stoettingsfjellet (Åsele Lappmark) (1 ♂, MZLU); *B. escheri* ♂, O.G., Bhm. (Östergötland = Ostra Gothia, Bohemann coll.) (3 ♂ NHRS).

*Atalanta hirtiloba* Speiser: **LECTOTYPE** (here designated in order to fix identity of the species) ♂ (Figure 9B), labelled (Figure 9A): “Ostpreussen/Mehlsachp./1.VIII.1922/P. Speiser”; “*Atalanta* (*Eucelidia*) *hirtiloba* Speiser”; “LECTOTYPE/*Atalanta* (*Eucelidia*)/*hirtiloba* Speiser/det. M. Ivković 2016” (NMW). **PARALECTOTYPE:** Same data as lectotype (1 ♀, NMW).

*Paramesia riparia* Robert: **LECTOTYPE** (here designated in order to fix identity of the species) ♀, labelled: “1400/36”; “MHNM, Paris/ED6073”; “LECTOTYPE [red label]”; “LECTOTYPE/of *Paramesia*/*riparia* Robert/des. B.J. Sinclair 1999 [red label] (MNHM). **PARALECTOTYPES:** same data as lectotype except, ED6074 (1 ♀, MNHM); same data as lectotype except, ED6075 (1 ♀, MNHM); same data as lectotype except, ED6076, *Paramesia ripara* (1 ♂, MNHM) (=*Wiedemannia pirata* Mik).

**Note on type specimens.***Wiedemannia zetterstedti*, *W. escheri* and *W. hirtiloba* were all described on the basis of an unspecified number of male and female specimens [34,35,36]. Although the lectotype of *P. riparia* is a female, it is confirmed as conspecific with *W. zetterstedti* on the basis of the brown-coloured face, wing shorter than 5 mm and erect costal setae appearing opposite the humeral crossvein. Images of the type specimens are available online: https://science.mnhn.fr/institution/mnhn/collection/ed/item/ed6073?listIndex=1&listCount=6 (accessed on 7 February 2022).

**Additional material examined. Bosnia & Herzegovina:** Miljacka, 3.vii.2012, M. Ivković (2 ♂, CNC). **Czech Republic:** Bohemia, Pařížov, J. Trakal (2 ♂, 1 ♀, CNC); Jeseník, Bělá River [ca. 50°6′54″ N 17°14′32″ E], 7–9.ix.1990, B.J. Sinclair (1 ♂, CNC). **Denmark:** Endrupholm, 18.vii.1920, P. Nielsen (1 ♂, 1 ♀, USNM). **Germany:** Ahrtal, Schuld, 250 m, 16–17.vi.2001, Ahr, B.J. Sinclair (3 ♂, 3 ♀, ZFMK); Bonn, Sieg, 25.v.2003, B.J. Sinclair (4 ♂, 1 ♀, ZFMK); Dachau, 18.vii.1913 (3 ♂, USNM); Moseltal, Zell, 11.viii.2001, Altlayer Bach, B.J. Sinclair (3 ♂, 1 ♀, ZFMK).

**Diagnosis.** This species is distinguished from other species of the *Wiedemannia zetterstedti* group by the following combination of characters: face brownish in both sexes, without stripe; wing usually shorter than 5 mm; stout erect setae on costa beyond humeral crossvein; clasping cercus brown, similar to epandrium, with lobes not in same plane and prominent anterior lobe.

**Redescription.** Wing length 3.4–4.8 mm. **Male** (Figure 10C). Head with gena, postgena and lower half of occiput with blue-grey pruinescence; gena less than half as deep as eye height. Face slightly wider than antennal sockets, dark, brownish (Figure 10A); distinct carina on lower margin. Frons short, brown, broader than face; ocellar triangle and vertex brown. Pair of long ocellar setae; one pair of strong vertical setae; 5–7 distinct upper postocular setae, all similarly sized and black, with interspersed shorter setae; lower postocular setae much finer and paler, merging with pale, longer setae on middle and lower occiput and postgena; several dark setulae on vertex and between ocellar area and eye margin. Antenna brown; scape and pedicel subequal; scape with dorsal setulae; pedicel with circlet of apical setulae; postpedicel pointed ovate; arista-like stylus longer than length of face.

Antepronotum with row of short setae, 1–2 slightly stronger. Proepisternum with some long, fine pale setae. Scutum brown, faintly bivittate; scutellum concolourous; postpronotal lobe and lower margin of notopleuron with faint blue-grey pruinescence. Pleura with dull blue-grey pruinescence. Mesonotum with 5 pairs of dorsocentral setae; several short acrostichal setulae, irregularly biserial, anterior to second dorsocentral seta; 1 postpronotal seta; 2 notopleural setae, usually with dark setulae on lower half; 1 presutural supra-alar seta, usually with several dark setulae; 0 postsutural supra-alar setae; 1 postalar seta; 1 pair scutellar setae with dark marginal setulae. Laterotergite with cluster of pale setae.

Wing membrane infuscate, veins dark; basal costa seta extending to humeral crossvein (Figure 10C). Origin of veins M_1_ and M_2_ widely separated at end of cell dm. Vein CuA + CuP short, faint streak or absent. Pterostigma narrow, elongate, faint to strong. Squama with pale fringes. Costal margin with short, strong erect setae beyond humeral crossvein. Halter with dark knob and paler shaft.

Legs entirely dark brown, except coxae with pale grey pruinescence; uniformly covered with rows of small dark setulae, slightly longer on ventral side of fore femur; all femora with strong anterior and posterior preapical seta; fore coxa with 2–3 long erect setae; hind tibia without erect, spine-like posterodorsal setae.

Abdomen concolourous with thorax, covered in short setae; lateral third of tergites and sternites with bluish pruinescence. Terminalia (Figure 3G,H and Figure 10B): Hypandrium subequal in length with epandrium, with bluish pruinescence, bearing 2−4 setae. Epandrium elongate, oval, covered with dark, long setae; rounded surstylus emerging from inner face apically. Clasping cercus brown, concolourous with epandrium, subequal to length of epandrium; bifurcated (bilobed), with lobes in different planes; prominent anterior lobe usually longer than posterior lobe, usually with longer outer setae; posterior lobe thumb-like with long setae along margin; inner face with peg-like setae on thumb-like part of clasping cercus. Phallus more or less linear, brownish; distiphallus straight, without swelling in middle.

**Female.** Similar to male except terminalia; cercus short ovate and minutely pilose.

**Distribution.** This species is widespread in Europe, recorded from Austria, Belgium, Bosnia & Herzegovina, Czech Republic, Denmark, Finland, France, Germany, Lithuania, Norway, Poland, Russia, and Sweden (Figure 11).

**Remarks.**  *The length, width and setation of the apical lobes of the clasping cercus in W. zetterstedti are highly variable (Figure 3F and Figure 10B), often in specimens captured in the same collecting event [37]. Despite this variation, features of the wing, face, and leg setation are consistent among specimens*.

## 4. Discussion

After a thorough morphological study of all available type material and additional material of all species that belong to the *Wiedemannia zetterstedti* group, as well as additional confirmation by molecular species concept, we can confirm with certainty that *W. escheri* and *W. hirtiloba*, as suggested by Niesiolowski [37], are synonyms of *Wiedemannia zetterstedti*. On the other hand, we confirm the statement from Collin [9] that Engel [23] was incorrect in synonymizing *W. longipennis* with *W. escheri,* and the former is a valid species of this group. *Wiedemannia ouedorum* is a junior synonym of *W. czernyi*. The subspecies *W. czernyi rufipes* is elevated to species level, and *W. ulrichi* sp. nov. is described as a new endemic species from Corsica (France).

The geographical distribution by country of all species is given in Figure 11. *Wiedemannia zetterstedti* is distributed in northern and central Europe [20], with patchy presence in the southern parts of France [38] and the central part of Bosnia and Herzegovina [39] (as *Wiedemannia* sp. A). *Wiedemannia longipennis* is present from Romania, through the Balkan Region and Greece to Turkey, Georgia, and Lebanon and is present in the northern and central part of Italy. *Wiedemannia czernyi* is present in Italy, Spain, southern France, and Northern Africa (Morocco and Algeria), while *W. rufipes* is presently known only from Romania, Greece, and Georgia.

## Data Availability

Data is contained within the article. The data presented in this study are available in this article.
